# Phosphodiesterase-5 inhibitors have distinct effects on the hemodynamics of the liver

**DOI:** 10.1186/1471-230X-9-69

**Published:** 2009-09-18

**Authors:** Leonie Halverscheid, Peter Deibert, René Schmidt, Hubert E Blum, Torsten Dunkern, Benedikt HJ Pannen, Wolfgang Kreisel

**Affiliations:** 1 Department of Anesthesiology, University Hospital Duesseldorf, Germany; 2 Department of Preventive and Rehabilitative Sport Medicine, University Hospital Freiburg, Germany; 3 Department of Anesthesiology and Critical Care Medicine, University Hospital Freiburg, Germany; 4 Department of Medicine II, University Hospital Freiburg, Germany; 5 Department of Biochemistry, Nycomed GmbH, Konstanz, Germany

## Abstract

**Background:**

The NO - cGMP system plays a key role in the regulation of sinusoidal tonus and liver blood flow with phosphodiesterase-5 (PDE-5) terminating the dilatory action of cGMP. We, therefore, investigated the effects of PDE-5 inhibitors on hepatic and systemic hemodynamics in rats.

**Methods:**

Hemodynamic parameters were monitored for 60 min. after intravenous injection of sildenafil and vardenafil [1, 10 and 100 μg/kg (sil1, sil10, sil100, var1, var10, var100)] in anesthetized rats.

**Results:**

Cardiac output and heart rate remained constant. After a short dip, mean arterial blood pressure again increased. Systemic vascular resistance transiently decreased slightly. Changes in hepatic hemodynamic parameters started after few minutes and continued for at least 60 min. Portal (var10 -31%, sil10 -34%) and hepatic arterial resistance (var10 -30%, sil10 -32%) decreased significantly (p < 0.05). At the same time portal venous (var10 +29%, sil10 +24%), hepatic arterial (var10 +34%, sil10 +48%), and hepatic parenchymal blood flow (var10 +15%, sil10 +15%) increased significantly (p < 0.05). The fractional liver blood flow (total liver flow/cardiac output) increased significantly (var10 26%, sil10 23%). Portal pressure remained constant or tended to decrease. 10 μg/kg was the most effective dose for both PDE-5 inhibitors.

**Conclusion:**

Low doses of phosphodiesterase-5 inhibitors have distinct effects on hepatic hemodynamic parameters. Their therapeutic use in portal hypertension should therefore be evaluated.

## Background

Nitric oxide (NO) plays a crucial role in hepatic microvascular blood flow under physiological conditions [1-5]. Hepatic vascular resistance is regulated on the one hand by contraction or relaxation of smooth muscle cells in the terminal arterioles. On the other hand, perisinusoidal stellate cells (Ito-cells) regulate sinusoidal tonus depending on concentration of NO synthesized by the sinusoidal endothelial cells. The diameter of liver sinusoids is responsible for up to 1/3 of the intrahepatic vascular resistance and is regulated by an interplay of endothelial cells, hepatocytes and stellate cells [6,7]. NO is synthesized by endothelial cells and activates soluble guanylate cyclase of stellate cells. This results in the formation of cGMP that regulates the tonus of stellate cells and sinusoids [8,9]. This action is terminated by phosphodiesterase-5 (PDE-5), which converts cGMP to 5'-GMP [10,11]. Furthermore, vascular tonus depends on the differential distribution of α- and β-receptors in the blood vessels. Angiotensin II and humoral factors, e.g., endothelins, with strong vasoconstrictor effects within extrasinusoidal and sinusoidal sites contribute to the regulation of liver blood flow [12-14]. Recently it was shown that induction of heme oxygenase-1 may reduce ischemia/reperfusion injury, probably by enhancing microvascular blood flow [15]. Data from the same group suggest an interplay between hepatic NO synthesis and heme oxygenase-1 regulation [16].

In liver cirrhosis, the NO - cGMP system is dysregulated. Portal hypertension is caused by an increased intrahepatic vascular resistance resulting from the disturbed liver architecture, perisinusoidal fibrosis, and cellular alterations of liver sinusoids as well as from functional changes. Due to a reduced activity of the endothelial NO synthase (eNOS) in liver endothelial cells NO decreases whereas hepatic stellate cells transform to contractile myofibroblasts [5,7,17-21]. These factors and an increased PDE-5 activity in liver cirrhosis result in the contraction of sinusoids [22-25]. In contrast to the intrahepatic condition in the splanchnic vascular system, NO production increases causing dilation of the mesenteric blood vessels and splanchnic hyperperfusion [5,26,27]. Apart from liver cirrhosis, an altered NO metabolism also occurs in other clinical settings, such as ischemia and reperfusion injury during liver surgery [1,28,29]. Several animal studies have shown that a selective modulation of NO metabolism in the liver reduces intrahepatic resistance and portal pressure in cirrhosis [30-36].

It is intriguing to investigate whether PDE-5 inhibitors which inhibit the conversion of cGMP to 5'-GMP could dilate hepatic sinusoids and increase hepatic blood flow. In a previous clinical pilot study we showed that the PDE-5 inhibitor vardenafil increases portal venous flow in normal and cirrhotic liver and lowers portal pressure and hepatovenous pressure gradient in cirrhotics [37]. In a patient with portopulmonary hypertension we could further demonstrate that the PDE-5 inhibitor tadalafil lowers both pulmonary arterial and portal pressure [38]. Recently, Lee et al. showed that after a standard dose of 50 mg sildenafil hepatic production of cyclic guanosine monophosphate increases leading to a significant decrease of hepatic sinusoid resistance (34). These authors found no change in HVPG [39]. Clemmesen et al. [40] observed a > 10% decrease of HVPG in 4 of 10 patients with liver cirrhosis. However, from animal experiments [41] and case reports [42-44] it was considered that PDE-5 inhibitors may even increase portal pressure.

The conflicting results obtained with PDE 5 inhibitors in the clinical setting require a thorough investigation in an experimental model prior to proceed to large scale clinical studies. Neither the optimal dose of PDE 5 inhibitors nor the optimal parameters of efficacy are known for a potential use of these drugs in liver cirrhosis. In this study we analyzed the effects of PDE-5 inhibitors on hemodynamics of normal liver in rats. We collected exact measurements of the effects of sildenafil and vardenafil, respectively, on hepatic blood flow and vascular resistances, portal venous pressure, and regional hepatic perfusion as well as systemic hemodynamic variables, e.g. cardiac output.

## Methods

### Reagents

Isoflurane was purchased from Abbott (Wiesbaden, Germany), pancuronium from Organon (BH Oss, Netherlands). Sildenafil and vardenafil were obtained from the Nycomed GmbH, (Konstanz, Germany). They were dissolved in 0.9% NaCl, containing 0.04% 0.1 N HCl.

### Animals

Overnight fasted male Sprague Dawley rats (Charles River, Sulzfeld, Germany) weighing 388 ± 37 g were used for all experiments. The experimental protocol was approved by the local Animal Care and Use Committee. All animals received care according to the Guide for the Care and Use of Laboratory Animals (American Association for Laboratory Animal Science. MD: NIH 1985).

### Animal preparation

After inhalational induction of anesthesia with isoflurane a tail vein was cannulated and a tracheotomy was performed. After muscle relaxation by intravenous injection of pancuronium (0.1 mg/kg i.v.) the animals were mechanically ventilated (Rodent Ventilator UB 7025-10, Harvard Apparatus, March-Hugstetten, Germany), under continued isoflurane anesthesia. For compensation of evaporative losses during the initial procedure of surgical preparation 4 ml/kg/h of a crystalloid solution (Jonosteril^®^, Fresenius, Bad Homburg, Germany) were continuously infused. An arterial line (polyethylene PE-50 tube) was placed into the left femoral artery for blood pressure monitoring and blood withdrawal. For cardiac output analysis by the transpulmonary thermodilution technique, a thermistor tip catheter (9490E, Columbus Instruments, Columbus, OH, USA) was inserted into the aortic arch through the left carotid artery. For monitoring central venous pressure and injection of saline at 4°C to measure cardiac output, a PE-50 catheter was positioned close to the right atrium via the right external jugular vein. At the time of laparotomy the continuous infusion of Jonosteril was increased from 4 to 10 ml/kg/h. Ultrasound flow probes were placed at the common hepatic artery and the portal vein (T206, small animal flow meter, Transonic, Ithaca, NY, USA). In addition, the portal vein was cannulated (26G, Insyte-W, BD, USA) to measure portal pressure. For microvascular (parenchymal) blood flow measurement, a microvascular flow probe (DP10M 100ST for DRT-4 Laser Doppler Monitor, Moor Instruments Ltd., Axminster, UK) was placed in a defined position on the surface of the left liver lobe. The body temperature was maintained normothermic (37 ± 0.5°C) throughout the experiment.

### Experimental Protocol

After stabilization for 15 min after surgery, baseline hemodynamic parameters were measured. Animals were then randomized into the following groups: sil100 100 μg/kg (n = 7), sil10 10 μg/kg (n = 7), sil1 1 μg/kg (n = 5), va100r 100 μg/kg (n = 6), var10 10 μg/kg (n = 7), var1 1 μg/kg (n = 5), controls 1 ml/kg 0.9% NaCl containing 0.04% 0.1 N HCl (n = 7). In order to minimize plasma volume related alterations of hemodynamic parameters the pharmacological intervention was carried out in a standard volume of 100 μl/kg injected over 90 seconds via the tail vein. Heart rate, mean arterial blood pressure, central venous pressure, portal venous pressure, portal venous flow, hepatic arterial flow, hepatic parenchymal flow and cardiac output were measured 0.5, 1, 3, 5, 10, 20, 30, 45 and 60 min after injection. From the data we calculated the "fractional liver flow": Arterial liver flow + portal liver flow/cardiac output. It represents the proportion of cardiac output passing through the liver.

### Data analysis

Normal distribution of all variables was tested before statistical analyses using the Kolmogorov-Smirnov test procedure. Changes between baseline and 60 min were analyzed using Wilcoxon rank sum test. Statistical differences between the groups at baseline were determined using a Kruskal-Wallis test on ranks. All p-values were two-sided and a p-value of < 0.05 was considered statistically significant. Analyses were performed using the SPSS software (version 15.0).

## Results

**Figure 1:**
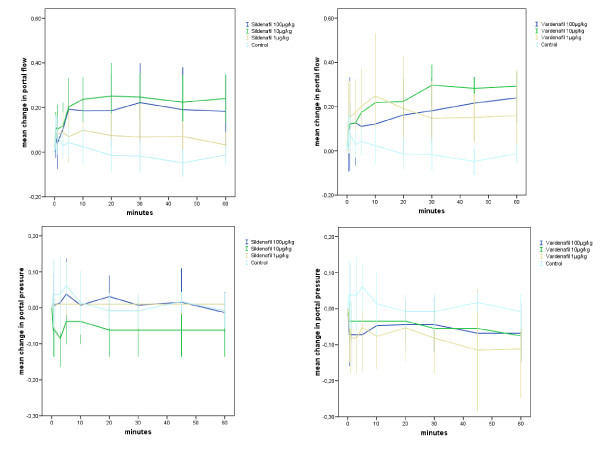
**Course of relative changes of portal flow and portal pressure after slow intravenous injection of different doses of sildenafil, vardenafil, or 0.9% NaCl**. Ordinate: time (minutes) Abscissa: relative change (e.g. 0.00 means baseline; 0.10 means: increase by 10%; -0.10 means: decrease by 10%). The values are indicated as mean ± 95% confidence intervals. Panel 1: Portal flow. Panel 2: Portal pressure. The values are indicated as mean ± 95% confidence intervals. The curves at different doses are marked by colours.

**Figure 2:**
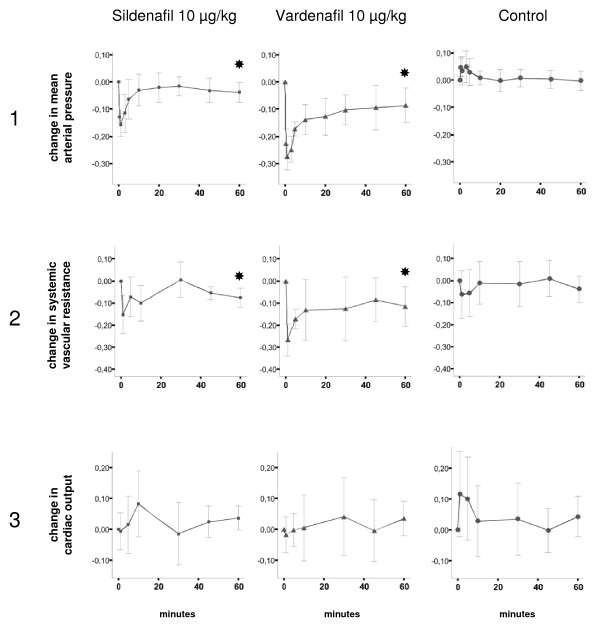
**Course of relative changes of systemic hemodynamic parameters after slow intravenous injection of 10 μg/kg sildenafil or vardenafil or 0.9% NaCl**. Ordinate: time (minutes). Abscissa: relative change (e.g. 0.00 means baseline; 0.10 means: increase by 10%; -0.10 means: decrease by 10%). The values are indicated as mean ± 95% confidence intervals. Significant changes at 60 min (p < 0.05; Wilcoxon rank sum test) are marked with black stars. Panel 1: Mean arterial pressure. Panel 2: Systemic vascular resistance. Panel 3: Cardiac output.

**Figure 3:**
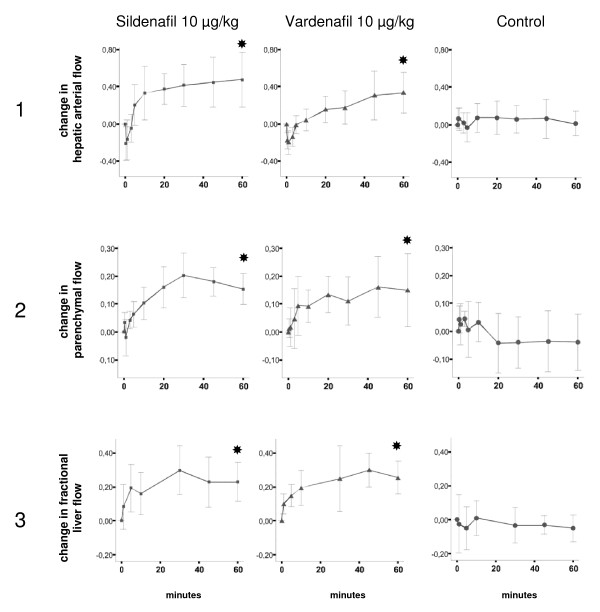
**Course of relative changes of hepatic hemodynamic parameters after slow intravenous injection of 10 μg/kg sildenafil or vardenafil or 0.9% NaCl**. Ordinate: time (minutes). Abscissa: relative change (e.g. 0.00 means baseline; 0.10 means: increase by 10%; -0.10 means: decrease by 10%). The values are indicated as mean ± 95% confidence intervals. Significant changes at 60 min (p < 0.05; Wilcoxon rank sum test) are marked with black stars. Panel 1: Hepatic arterial flow. Panel 2: Parenchymal flow. Panel 3: Fractional liver flow.

The effects of sildenafil and vardenafil on hemodynamic parameters are presented in Table 1, Figure 1, Figure 2, and Figure 3. Table 1 shows data at baseline and 60 min after injection of sildenafil or vardenafil at the three concentrations, compared to controls. The 60 min time point was chosen because preliminary data had demonstrated that after 60 min a steady state is reached. Figure 1 depicts the time-dependent course of the two most important hepatic hemodynamic parameters, portal flow and portal pressure, at the three different doses of sildenafil and vardenafil. These curves show 1. that the 10 μg/kg doses seem to induce the most impressive increase of portal blood flow, and 2. that despite increasing portal flow the portal pressure does not increase. Therefore, we depict the courses of further systemic and hepatic hemodynamic variables at this dose in Figure 2 and 3.

**Table 1:** Baseline parameters and relative changes after 60 min, expressed as % of the baseline value

parameter	control		sildenafil 100 μg/kg		sildenafil 10 μg/kg		sildenafil 1 μg/kg		vardenafil 100 μg/kg		vardenafil 10 μg/kg		vardenafil 1 μg/kg	
**MAP** (mmHg)	84.19 [80.3-88.0]	-0.3 [-3.8-3.2]	85.0 [80.2-89.7]	-2.6 [-6.5-1.2]	84.9 [81.7-88.2]	**-3.9 **✸ [-7.6 - -0.0]	83.9 [81.2-86.5]	**1.1 **✸ [0.4-1.9]	82.9 [79.7-86.2]	-7.4 [-16.3-1.4]	80.9 [77.5-84.3]	**-8.7 **✸ [-15.0 - -2.4]	88.0 [82.5-93.5]	**-11.0 **✸ [-16.7 - -5.3]
**HR** (1/min)	366.4 [348.8-384.1]	-1.1 [-2.9-0.7]	375.0 [351.0-399.0]	-1.9 [-3.0-6.7]	375.7 [364.3-387.2]	3.2 [-0.0-0.1]	366.0 [332.2-399.8]	2.9 [-2.1-3.9]	368.3 [353.3-383.4]	2.4 [-1.3-6.2]	362.9 [353.3-372.4]	5.2 [-3.4-13.7]	346.0 [324.3-367.7]	1.3 [-3.4-6.0]
**SVR** (mmHg/ml·min)	0.7 [0.6-0.8]	-4.0 [-10.1-2.1]	0.7 [0.61-0.77]	-2.0 [-7.8-3.8]	0.8 [0.68-0.90]	**-7.7 **✸ [-12.1 - -3.3]	0.8 [0.7-0.9]	-0.7 [-8.5-7.1]	0.7 [0.6-0.8]	**-8.7 **✸ [-13.6 - -3.8]	0.7 [0.5-0.8]	**-11.6 **✸ [-20.6 - -2.7]	0.7 [0.5-0.8]	**-11.6 **✸ [-22.1 - -1.1]
**CO** (ml/min)	116.7 [98.4-135.0]	4.4 [-2.2-10.9]	120.3 [108.6-131.9]	-0.8 [-4.5-2.9]	106.7 [90.0-123.4]	3.7 [-0.1-7.5]	104.1 [85.33-122.9]	2.3 [7.0-11.6]	113.0 [100.0-126.0]	1.3 [-3.8-6.4]	119.7 [99.1-140.4]	3.5 [-2.0-9.1]	127.5 [103.8-151.2]	0.4 [-6.0-6.9]
**A hep Resist** (mmHg/ml·min)	20.8 [15.9-25.8]	0.8 [-13.9-15.5]	20.3 [14.8-25.8]	**-17.4 **✸ [-32.8 - -2.1]	25.1 [19.3-30.9]	**-32.2 ✸ ** [-47.8 - -16.7]	23.3 [16.1-30.5]	-1.8 [-6.9-3.2]	23.4 [18.6-28.2]	**-21.8 ✸ ** [-35.2 - -8.4]	20.7 [16.5-24.9]	**-30.1 **✸ [-41.2 - -18.9]	16.9 [13.5-20.2]	**-18.8 **✸ [-28.1 - -9.5]
**A hep Flow** (ml/min)	4.1 [3.2-5.1]	0.9 [-12.0-13.9]	4.4 [3.1-5.6]	21.3 [-0.0-42.6]	3.5 [2.6-4.4]	**47.5 **✸ [17.8-77.2]	3.7 [2.7-4.7]	3.3 [-2.6-9.1]	3.5 [2.8-4.3]	**20.2 **✸ [3.2-37.2]	3.9 [3.2-4.6]	**33.5 **✸ [11.6-55.5]	5.1 [4.2-6.1]	9.6 [-5.4-24.7]
**Port Resist** (mmHg/ml·min)	0.2 [0.1-0.2]	2.8 [-1.9-7.5]	0.1 [0.11-0.14]	-20.3 [-34.5 - -6.2]	0.1 [0.1-0.2]	**-33.8 **✸ [-46.8 - -20.8]	0.2 [0.1-0.2]	0.8 [-7.0-8.5]	0.1 [0.1-0.2]	**-22.0 **✸ [-34.0 - -10.0]	0.2 [0.1-0.2]	**-31.0 **✸ [-40.6 - -21.4]	0.2 [0.1-0.2]	**-38.6 **✸ [-61.1 - -16.0]
**Port Flow** (ml/min)	23.3 [20.1-26.5]	-1.3 [-0.5-2.9]	25.0 [21.7-28.3]	**18.3 **✸ [2.0-34.5]	25.3 [22.9-27.7]	**24.1 **✸ [13.3-34.9]	21.7 [17.6-25.7]	3.3 [-2.4-8.9]	22.7 [20-6 - 24-8]	**23.9 **✸ [12.5-35.4]	24.1 [21.1-27.1]	**29.2 **✸ [22.5-35.9]	24.0 [21.1-26.9]	15.9 [-4.6-36.5]
**Parench Flow** (ml/min)	135.2 [121.0-149.4]	-3.9 [-14.0-6.2]	151.8 [130.5-173.0]	**12.4 **✸ [2.5-22.2]	133.7 [115.4-151.9]	**15.3 **✸ [9.8-20.8]	131.3 [122.2-140.5]	**6.3 **✸ [-1.9-14.5]	126.3 [103.2 -.149.4]	**28.8 **✸ [10.4-47.3]	146.2 [118.2-174.1]	**15.0 **✸ [1.8-28.2]	130.1 [114.1-146.0]	6.8 [-10.0-23.6]
**Port Pressure** (mmHg)	6.3 [5.7-6.9]	-0.8 [-5.7-4.1]	6.1 [5.5-6.7]	-1.3 [-6.9-4.3]	6.1 [5.9-6.3]	-6.2 [-13.6-1.3]	5.7 [4.8-6.6]	1.0 [-1-8 - 3-9]	6.3 [5.8-6.7]	-6.8 [-15.0-1.3]	6.4 [5.7-7.1]	-7.5 [-14.2 - -0.1]	6.3 [5.3-7.4]	-11.2 [-24.8-2.4]
**Fract Liver Flow** (%)	23.8 [20.6-27.0]	-4.8 [-12.6-3.0]	24.5 [21.7-27.3]	**19.3 **✸ [5.6-32.9]	27.5 [23.7-31.3]	**22.9 **✸ [11.6-34.2]	24.5 [19.5-29.6]	1.4 [-8.6-11.4]	23.6 [19.2-28.0]	**22.0 **✸ [10.9-33.2]	23.7 [21.3-26.0]	**25.5 **✸ [15.9-35.1]	23.3 [18.1-28.5]	14.5 [-3.7-32.7]

Significant changes (p < 0.05; Wilcoxon rank sum test) are marked with ✸. MAP = mean arterial pressure (mm Hg), HR = heart rate (1/min), SVR = systemic vascular resistance (mm Hg·ml^-1^·min^-1^), CO = cardiac output (ml/min), A Hep Resist = hepatic arterial resistance (mm Hg·ml^-1^·min^-1^), A Hep Flow = hepatic arterial flow (ml/min), Port Resist = portal venous resistance (mm Hg·ml^-1^·min^-1^), Port Flow = portal venous flow (ml/min), Parench Flow = parenchymal flow (ml/min), Port Pressure = portal venous pressure (mm Hg). Fract Liver Flow = fractional liver flow (arterial liver flow + portal liver flow/cardiac output) (%).

### Effect of sildenafil and vardenafil on systemic hemodynamic parameters

In the control group, no parameter showed a significant change as compared to baseline (Table 1). Sil and var at 1 μg/kg and 10 μg/kg induced a slight but significant change of mean arterial pressure (MAP) by 1.1 - -11.0%. Systemic vascular resistance (SVR) decreased significantly in the sil10 and all vardenafil groups by 7.7 - 11.6%. Most importantly, in none of the groups heart rate or cardiac output changed significantly, there was a short initial increase of heart rate and cardiac output after injection in all of the groups. Taken together, sildenafil and vardenafil induced only minor changes in systemic hemodynamic parameters.

### Effect of sildenafil and vardenafil on hepatic hemodynamic parameters

Hepatic arterial resistance decreased by 17.4 - 32.2% in all intervention groups except in sil1. Consistent with these findings hepatic arterial blood flow increased in all intervention groups, reaching statistical significance in the sil10 (increase by 47.5%), var10 and var100 groups (increase by 33.5% and 20.2%), respectively. Portal resistance (calculated as portal pressure minus central venous pressure divided by portal flow) significantly decreased in var1, var10, var100, and sil10 groups by 22.0 - 38.6%. Portal venous flow increased significantly with sil and var at 10 and 100 μg/kg, respectively. Sil and var at 10 ug/kg increased portal flow by 24.1% and 29.2%, respectively. Hepatic parenchymal blood flow significantly increased in all sil groups (by 6.3 - 15.3%) and in the var10 and var100 groups (by 15.0% and 28.8%). Taken together, in animals with normal liver we observed a decrease of arterial hepatic and portal (transhepatic) resistance and an increase of portal, arterial and parenchymal blood flow of the liver.

Most importantly, in none of the intervention groups portal pressure increased. To the contrary, in sil10, sil100, var1, var10, and var100 portal pressure decreased and nearly reached statistical significance in the var10 group (p = 0.06).

Additional information can be derived from Figure 2 and Figure 3. Immediately after injection of sil or var at 10 μg/kg MAP decreased (Figure 2, panel 1) but increased again only a few min later without reaching baseline levels at 60 min. This pattern was observed in all intervention groups. SVR also dropped and then gradually increased without complete normalization at the end of the experiment (Figure 2, panel 2). PDE-5 inhibitors had no effect on the heart rate (data not shown) or cardiac output (Figure 2, panel 3).

Already a few minutes after the injection of sil or var hepatic arterial flow (Figure 3, panel 1), hepatic parenchymal flow (Figure 3, panel 2), and portal venous flow (Figure 1) increased. This increase was independent from cardiac output (which remained constant) and the slight drop of MAP. The hepatic effects became even more pronounced with time, e. g., after 15 min when drug distribution should have reached a steady state. Despite increased portal venous and hepatic arterial blood flow portal pressure does not increase. On the contrary, there is a trend to a decrease (Figure 1). From the data we calculated the fractional liver flow (portal venous + hepatic arterial blood flow/cardiac output = proportion of cardiac output passing through the liver, Figure 3, panel 3). In sil10 it increased at 10, 30, and 60 min by 16%, 30%, and 23%. In var10 it increased by 14%, 24%, and 26%.

## Discussion

This is the first experimental study in which the effect of different low doses of two inhibitors of phosphodiesterase 5 on hepatic and systemic hemodynamics was investigated up to 60 min after intravenous injection. We showed a distinct effect of the PDE-5 inhibitors, sildenafil and vardenafil, at low doses on the hemodynamics of the liver: 1. Portal venous, hepatic arterial, and hepatic parenchymal blood flow increased. 2. Portal venous and hepatic arterial resistance decreased. 3. Portal pressure showed a trend towards decreasing. 4. Sildenafil decreases mean arterial pressure by <4% and vardenafil by ≤ 11%. 5. Heart rate, central venous pressure and cardiac output remained constant. 6. Fractional liver blood flow increased. Combining the data from Figure 1 and Table 1 we may conclude, that the 10 μg/kg dose is optimal for a more or less selective effect on hepatic hemodynamics, because it only slightly affects systemic hemodynamics parameters. These results contribute to the understanding of the physiology of liver hemodynamics and have potential implications for the treatment of portal hypertension.

About 25% of the cardiac output passes through the liver. Liver blood supply is provided by the hepatic artery (25 - 30%) and the portal vein (up to 75%) [3-5]. Blood flow rates in both vessels closely correlate and are regulated by multiple factors. A decline in portal venous blood flow enhances hepatic arterial flow by reducing hepatic arterial vascular resistance and vice versa. This mechanism known as hepatic arterial buffer response maintains the total liver blood supply [45].

Our data show that PDE-5 inhibitors - after intravenous application - increase both arterial and portal liver blood flow. The local vasodilation induced by these drugs abolishes - at least in this experimental setting - the hepatic buffer response. However, this effect is more than compensated by the decrease of portal resistance. This is reflected by the fact that the parenchymal liver flow is increased by >15% and that the portal pressure does not increase but rather shows a tendency towards decrease (almost significant in the var10 group). Even if there is a dilation of splanchnic vessels leading to an increased blood flow towards the liver, all effects of PDE-5 inhibitors on extrahepatic arterial vessels are more than compensated by their intrahepatic effects. There are conflicting data whether or not an increase of portal flow alone increases portal pressure. Lee et al. [46] clearly demonstrated that a postprandial increase of portal flow leads to an increase of portal pressure even in normal liver. This increase was much more pronounced in cirrhotics. However, even in cirrhotic liver organic nitrates can modulate the postprandial increase of portal pressure [47]. Jiao et al. [48] observed a dramatic increase of portal pressure following increase of portal flow in the isolated perfused porcine liver. Recently, Zipprich et al. [49] investigated the effect of a selective increase of flow in the hepatic artery or the portal vein on portal pressure in normal and cirrhotic rat liver. Their system with in-situ perfused rat liver is in many respects comparable to our model. They found, that an increase of arterial perfusion increases portal pressure both in normal and in cirrhotic liver, whereas an increase of portal flow increases portal pressure only in cirrhotic liver. Despite an increase of both arterial and portal hepatic perfusion we observed no increase of portal pressure. Therefore, our data suggest that PDE-5 inhibitors act on intrahepatic structures beyond the point where portal and arterial flows merge. It may be speculated that even low levels of PDE-5 inhibitors increase the local cGMP concentration in the stellate cells and induce a dilation of the sinusoids.

There are only few papers which dealt with hemodynamic effects of PDE-5 inhibitors in animals or humans with cirrhosis [50-52]. However, these authors focused on systemic or renal effects, the hepatic hemodynamics were not investigated. In addition, the authors used very high doses of the PDE-5 inhibitors.

Loureiro-Silva et al. investigated the effect of sildenafil on hepatic hemodynamics in normal and cirrhotic rat liver [23]. They found an increased expression of both PDE-5 and soluble guanylate cyclase in cirrhotic liver. For functional studies they used perfused liver, methoxamine was used for preconstriction of the intrahepatic circulation. Endogenous NO production was inhibited by the NO synthase inhibitor L-NMMA (N-monomethyl-L-arginine) and vasodilation was induced by the NO donor SNAP (S-Nitroso-N-Acetylpenicillamine). In this setting a reduced vasodilatory response to SNAP in cirrhosis could be demonstrated, which is corrected (or even overcompensated) by 10^-8 ^M sildenafil. While this approach yields information on the regulation of the sinusoidal tonus, it is difficult to translate these data to patients with portal hypertension.

Our data partially confirm the data obtained by Colle et al. [41] who studied the effect of sildenafil on mesenteric blood flow, mean arterial pressure, and portal venous pressure at doses ranging from 10 - 10,000 μg/kg injected into the mesenteric artery or intravenously. In contrast to our experimental protocol, the authors monitored the parameters for up to 10 min after the injection only and used repetitive doses after 10 min washout. In this experimental setting it was not possible to detect the changes in hemodynamic parameters that we describe. Nevertheless, they also found the transient dip in arterial blood pressure after injection of the drug. At the lowest doses of 10 and 100 μg/kg given intravenously the relative decrease of mean arterial pressure was about 3%. The relative increase of mesenteric blood flow was ≤ 10%, and no change of portal venous pressure was observed. The initial drop of MAP and SVR in our study occurred presumably due to a transiently higher drug concentration immediately after intravenous injection. From the results obtained with high doses (1000 and 10000 μg/kg given intraarterially or intravenously) used in the study by Colle and coworkers no conclusions can be drawn whether or not PDE-5 inhibitors may be a risk for variceal bleeding in liver cirrhosis.

The present study in rats with normal liver demonstrates the influence of low-dose PDE-5 inhibitors on hepatic blood flow. In this setting they marginally interfere with systemic hemodynamics. With regard to cardiac output we calculated the values for fractional hepatic blood flow after drug injection (Figure 3, panel 3). These results revealed an increase in selective liver blood supply compared to baseline by 20 - 30%. To the best of our knowledge to date this has not been shown for any other drug. In our animal study the ideal dose for short term use is in the range of 10 μg/kg. This is much less than the standard dose of vardenafil (10 mg) or sildenafil (50 mg) used for therapy of erectile dysfunction in the clinical setting. With a dose in the range of 10 μg/kg the systemic hemodynamic effects of PDE-5 inhibitors could be neglected.

Our data are well in line with those of Lee et al [39]. These authors described an increase of cGMP levels in liver veins of cirrhotics after oral administration of 50 mg sildenafil but not in peripheral veins leading to a significant decrease of sinusoidal resistance but not of peripheral vascular resistance. Therefore it can be concluded that conditions may exist (oral vs. intravenous applications, low doses) in which hemodynamic effects of PDE 5 inhibitors prevail in the liver leaving systemic circulation more or less unaffected. Comparing oral administration to intravenous application, a suspended arterial buffer response was demonstrated in rats in this study. In healthy and cirrhotic human livers, we have shown that after oral administration the buffer response stays intact [37].

Our findings suggest a distinct effect of PDE-5 inhibitors on hepatic hemodynamics. However, we cannot definitively answer the question whether or not PDE-5 inhibitors can be used to treat portal hypertension (or other conditions such as ischemia/reperfusion injury). We are presently examining the responses to low doses of PDE-5 inhibitors in animals with portal hypertension. If PDE-5 inhibitors are beneficial in this model, patients with liver cirrhosis should be evaluated. The measures of efficacy should be the hepatovenous pressure gradient and clinical end points, such as bleeding or re-bleeding from esophageal varices.

## Conclusion

PDE-5 inhibitors at low doses have distinct effects on liver hemodynamics. Portal flow and parenchymal flow increase without enhancement of portal pressure in normal rat liver. The compromised NO-bioavailability in the cirrhotic liver leads to a constriction of the sinusoids, which contributes to the functional component of portal hypertension. It may be anticipated that this effect can be reversed by application of PDE-5 inhibitors.

## Abbreviations

PDE-5: phosphodiesterase-5; NO: nitric oxide; cGMP: cyclic guanosine monophosphate; sil: sildenafil; var: vardenafil.

## Competing interests

The University of Freiburg holds a European patent: Use of inhibitors of phosphodiesterase 5 for therapy or prophylaxis of portal hypertension.

## Authors' contributions

PD and WK had the idea to perform this study and conceived its details. LH and RS performed the experiments and contributed to the concept of the study. PD and WK wrote the manuscript, PD did the statistical evaluations. HEB participated in writing the manuscript and coordination of the study. TD provided us the drugs and participated in the concept of the study. BJP participated in the concept of the study. LH and PD contributed equally to the work. All authors read and approved the final manuscript.

## Pre-publication history

The pre-publication history for this paper can be accessed here:

http://www.biomedcentral.com/1471-230X/9/69/prepub
